# Extracellular glucose is crucially involved in the fate decision of LPS-stimulated RAW264.7 murine macrophage cells

**DOI:** 10.1038/s41598-020-67396-6

**Published:** 2020-06-29

**Authors:** Toshihiko Aki, Takeshi Funakoshi, Kanako Noritake, Kana Unuma, Koichi Uemura

**Affiliations:** Department of Forensic Medicine, Graduate School of Medical and Dental Sciences, Tokyo Medical and Dental University, 1-5-45 Yushima, Bunkyo-ku, Tokyo, 113-8519 Japan

**Keywords:** Cell death, Immune cell death

## Abstract

Pyroptosis, a type of inflammatory cell death, is dependent on the inflammatory caspase-mediated cleavage of gasdermin D (GSDMD), and the subsequent pore formation on plasma membranes through which interleukin (IL)-1β and IL-18 are released from cells. During proinflammatory activation, macrophages shift their metabolism from aerobic oxidative phosphorylation to anaerobic glycolysis. Hypoxia-inducible factor (HIF)1α is involved in the induction of IL-1β gene expression as well as the metabolic shift towards glycolysis. However, the relationships between pyroptosis and glycolysis, as well as between pyroptosis and HIF1α are poorly investigated. Here we show that lipopolysaccharide (LPS) stimulation of RAW264.7 murine macrophage cells results in pyroptosis when cells are cultured in high glucose medium. During pyroptosis, HIF1α activation occurs transiently followed by downregulation to sub-basal levels. HIF1α downregulation and pyroptosis are observed when cells are stimulated with LPS under high glucose conditions. We also found that intracellular levels of methylglyoxal (MGO), a side product of glycolysis, increase when cells are stimulated with LPS under high glucose conditions. The addition of glycolysis inhibitor and rapamycin suppresses HIF1α downregulation and pyroptosis. These results show that glycolysis plays a crucial role not only in pro-inflammatory activation, but also in pyroptosis in LPS-stimulated RAW264.7 macrophages.

## Introduction

Immune cell activation, which often results in a lytic form of cell death called pyroptosis, is critical for host defense against pathogen invasions^1^. Upon stimulation with lipopolysaccharides (LPS), which are components of the cell walls of gram-negative bacteria, macrophages are activated in a proinflammatory direction via the activation of inflammatory caspases. Caspase-1 is an inflammatory caspase involved in both the maturation and secretion of pro-inflammatory interleukin-1β (IL-1β) and IL-18 through plasma membrane pores^2–4^. A gasdermin family protein, gasderminD (GSDMD), has been shown to be responsible for pore formation on the plasma membrane. GSDM family proteins comprise N-terminal and C-terminal domains as well as a linker connecting them. GSDMD cleavage in its linker by inflammatory caspases results in the release of the N-terminal domain (p30). GSDMD-p30 translocates to the plasma membrane where it assembles to form pores through which IL-1β and IL-18 are secreted from the cell^5–11^.

It has been reported that hyperglycemia is a risk factor for many illnesses including ischemic cardiovascular injuries^12^, renal diseases^13^, and sepsis^14^. One of the detrimental effects of hyperglycemia on such diseases and injuries is its effect on inflammation. In response to stimulation by endotoxin and by bacterial as well as viral infection, a macrophage switches its metabolism from oxidative phosphorylation to glycolysis^15^. Thus, an increased dependence on glycolysis by proinflammatory macrophages is one of the mechanisms responsible for the deterioration of inflammation under hyperglycemic conditions. Hypoxia-inducible factor1 (HIF1), a transcriptional complex required for cell adaptation under low oxygen conditions, is essential for this metabolic shift from aerobic to anaerobic glycolysis^16^. In addition to its roles in metabolic shift, HIF1 is also involved in the induction of IL-1β gene expression. Upon formation of a transcriptional activation complex with pyruvate kinase M2 (PKM2), HIF1 binds to a hypoxia response element (HRE) on the IL-1β gene promoter, and induces the expression of its gene^17,18^.

Here we show that LPS stimulation of RAW264.7 macrophage cells cultured in high glucose medium leads to pyroptosis, which can be suppressed by a glycolytic inhibitor as well as rapamycin, an inhibitor of the mechanistic target of rapamycin (mTOR). Prior to pyroptosis, HIF1 is downregulated below basal levels. These results indicate the essential roles of glycolysis in endotoxin-induced pyroptosis of macrophages.

## Results

### LPS induces pro-inflammatory cytokine and M1 marker expression in RAW264.7 cells

**Figure 1:**
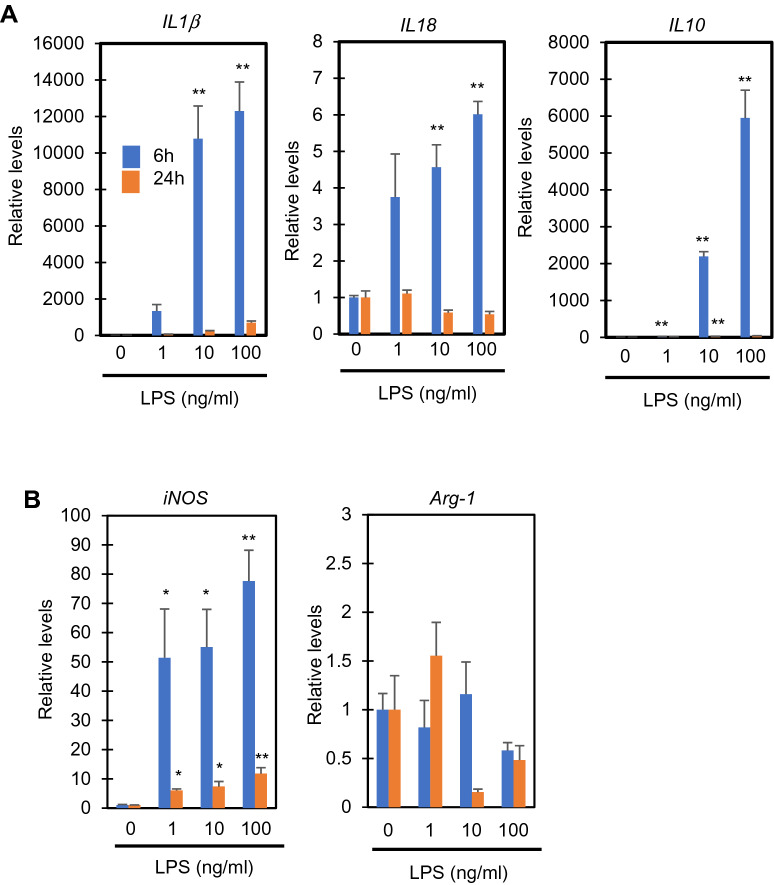
Expressions of cytokines and phenotypic markers in RAW264.7 cells stimulated by LPS. (**A**) The gene expressions of pro- and anti-inflammatory cytokines in response to LPS. The cells were treated with the indicated concentrations of LPS for 6 and 24 h, and qPCR analysis was performed to evaluate the levels of IL-1β, IL-18, and IL-10 relative to GAPDH. (**B**) M1 and M2 phenotypic marker expressions in response to LPS. The cells were treated with the indicated concentrations of LPS for 6 and 24 h, and qPCR analysis was performed to evaluate the levels of iNOS and arginase-1 (Arg-1) relative to GAPDH. The data represent means and S.E., n = 3 or 4, *P < 0.05, **P < 0.01 versus 0 ng/ml LPS (one-way ANOVA followed by Dunnett’s post-hoc multiple comparison test).

RAW264.7 cells cultured in high glucose (4.5 g/l) medium were stimulated with increasing concentrations (1, 10, and 100 ng/ml) of LPS for 6 and 24 h. qPCR analysis was performed to evaluate the gene expressions of pro- and anti-inflammatory cytokines. The expressions of pro-inflammatory IL-1β and IL-18 as well as anti-inflammatory IL-10 were induced by LPS stimulation for 6 h, and tended to return to basal levels after 24 h (Fig. 1A), suggesting that LPS has a mixed or biphasic effect on the inflammatory activation of cells as reported previously^19^. Immunoblot analysis of the intracellular and extracellular levels of IL-1β and IL-18 proteins showed that the proteins first increase intracellularly, and are then secreted from the cells in response to LPS stimulation (Supplementary Fig. 1). In addition, most of the IL-1β and IL-18 released from the cells are in the pro-forms, suggesting that LPS alone causes the increase and release of IL-1β and IL-18, but does not convert them to their active forms sufficiently (Supplementary Fig. 1). These observations are consistent with previous reports, showing that caspase-1 activation is scarcely observable in RAW264.7 cells due to the lack of an inflammasome component, apoptosis-associated speck-like protein contains a carboxyl-terminal CARD (ASC)^20,21^. The expression of a marker of pro-inflammatory M1 macrophages (classically activated macrophages), the inducible NO synthase (iNOS) gene, was significantly increased after LPS stimulation for 6 and 24 h (Fig. 1B). In contrast, the expression of a marker of anti-inflammatory M2 macrophages (alternatively activated macrophages), arginase-1 (arg-1), was somewhat decreased by stimulation with 10–100 ng/ml LPS for 24 h (Fig. 1B). Taken together, these results show that pro-inflammatory responses take place in LPS-stimulated RAW264.7 cells, although an anti-inflammatory reaction appears to occur during the early stage.

### LPS-induced pyroptosis is accompanied by extracellular acidosis in RAW264.7 cells

**Figure 2:**
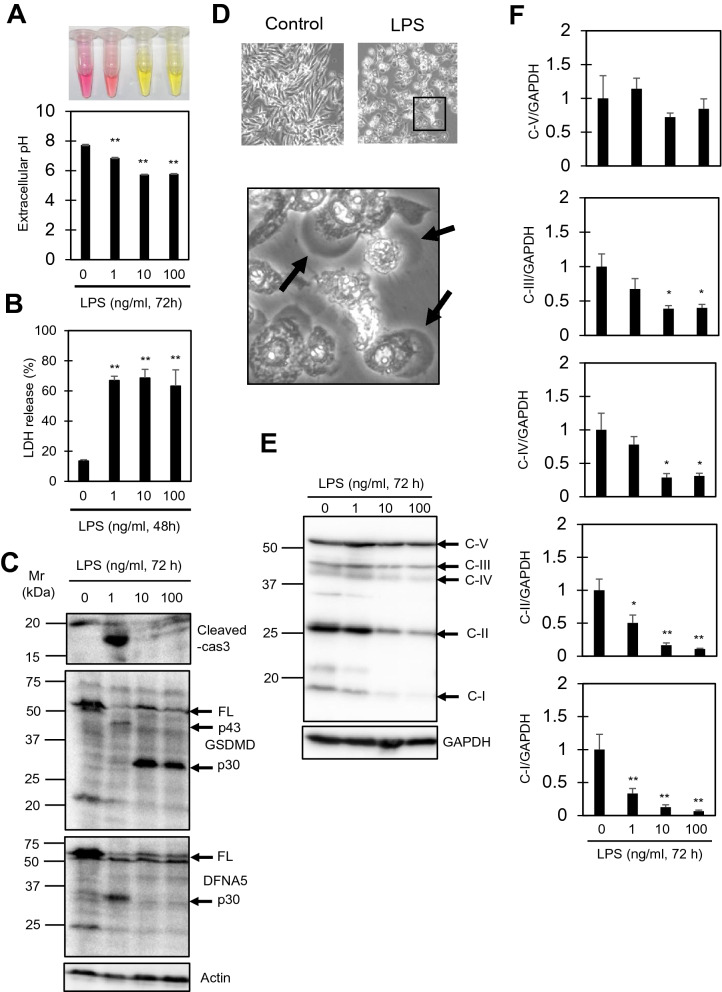
Pyroptosis in LPS-stimulated RAW264.7 cells is accompanied by extracellular acidosis as well as downregulation of mitochondrial respiratory proteins. (**A**) Extracellular pH (medium pH) of cells treated with the indicated concentrations of LPS for 72 h. Upper panel shows the color of the medium. (**B**) LDH release assay from cells treated with the indicated concentrations of LPS for 48 h. (**C**) Immunoblot analysis of cleaved-caspase3, GSDMD, and DFNA5 in cells treated with the indicated concentrations of LPS for 72 h. FL, full length. (**D**) Representative phase contrast images of cells stimulated with or without LPS (10 ng/ml, 48 h). The lower panel is an enlarged image of the framed portion of the upper right image. Arrows indicate ballooning of the plasma membrane. (**E**, **F**) Immunoblot analysis of mitochondrial respiratory proteins (OXPHOS proteins) in cells treated with the indicated concentrations of LPS for 72 h (**E**). C-I ~ V indicate proteins of mitochondrial respiratory complex I (NDUFB8), II (SDHB), III (UQCRC2), IV (MTCO1), and V (ATP5A). Levels of C-I ~ V to relative to GAPDH are shown in (**F**). All data represent means and S.E., n = 3 or 4, *P < 0.05, **P < 0.01 versus 0 ng/ml LPS (one way ANOVA followed by Dunnett’s post-hoc multiple comparison test).

The activation of macrophages in the pro-inflammatory direction is associated with a metabolic polarization into glycolysis, and the increased glycolytic rate was observed as an increase in extracellular acidification. In addition, macrophage activation often leads to subsequent cell death through pyroptosis, during which GSDMD cleavage releasing the p30 fragment is observed. We thus examined these two events after LPS stimulation for 72 h. A concentration-dependent decrease in the extracellular pH, which reached around 5.7 after 10–100 ng/ml LPS stimulation for 72 h, was observed when the cells were cultured in high glucose medium (Fig. 2A). The decrease in extracellular pH saturated at around 10 ng/ml LPS (Fig. 2A). LDH release assays showed that cell death was already observed at concentrations as low as 1 ng/ml LPS (Fig. 2B). Immunoblot analysis showed intracellular LDH levels to be almost constant at 24 h and rather decreased at 72 h of LPS stimulation, justifying the use of the LDH release assay as a cell death assay (Supplementary Fig. 2). Immunoblot analysis showed GSDMD cleavage to p30 in cells stimulated with 10–100 ng/ml LPS. In contrast, cleaved caspase3 was observed only in cells stimulated with 1 ng/ml LPS (Fig. 2C). Caspase3 activation in 1 ng/ml LPS-stimulated cells was further confirmed by the presence of the p43 and p30 fragments of GSDMD and DFNA5 (GSDME), respectively, which have been shown to be caspase-3-dependent fragments^22–24^ (Fig. 2C). Thus, cells stimulated with 1 ng/ml LPS seemed to undergo apoptosis, which should be followed secondary necrosis. Morphological analysis by microscopy indicated plasma membrane ballooning, a characteristic feature of pyroptosis, in cells stimulated with 10 ng/ml LPS (Fig. 2D). These results indicate an LPS concentration-dependent change in cell death from apoptosis to pyroptosis, and show that 10 ng/ml LPS is sufficient to induce the phenotypic polarization into oxygen-independent glycolysis and subsequent pyroptosis. Interestingly, we observed significant downregulation of OXPHOS proteins (complex I–IV) in cells treated with 10 or 100 ng/ml LPS for 72 h, further supporting the metabolic shift towards glycolysis in LPS-stimulated cells (Fig. 2E, F).

### HIF1α activation is followed by its downregulation to below basal level in RAW264.7 cells stimulated with LPS

**Figure 3:**
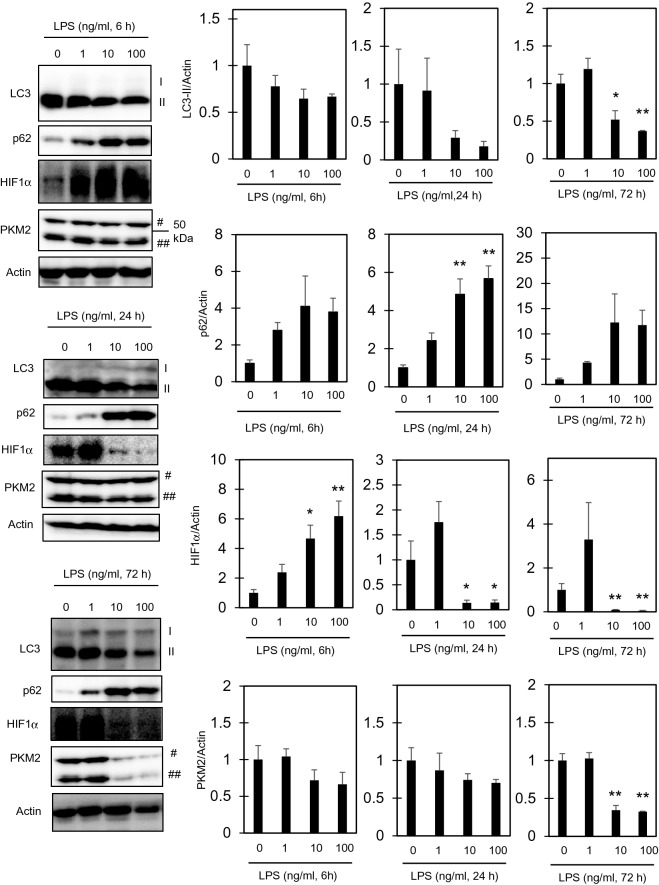
Levels of autophagy markers and HIF1α protein in RAW264.7 cells after treatment with LPS. The cells were treated with the indicated concentrations of LPS for 6, 24, and 72 h. Total cell lysates were extracted, and the levels of the indicated proteins (LC3, p62, HIF1α and PKM2) were determined by immunoblotting. #, a band corresponding to the molecular weight of PKM2 (~ 60 kDa) was used to quantify PKM2. ##, a smaller band (~ 40 kDa), which showed essentially the same behavior as the 60 kDa band, was also observed in RAW264.7 cells. Actin was also measured and used as an internal standard. The data represent means and S.E., n = 3 or 4, *P < 0.05, **P < 0.01 (one way ANOVA followed by Dunnett’s post-hoc multiple comparison test).

We next examined autophagy and HIF1α, both of which play crucial roles in the activation of macrophages^16,25^, in RAW264.7 cells stimulated with LPS for 6, 24, and 72 h. At every time point tested, LC3-II tended to decrease and p62 to increase in cells stimulated with 10–100 ng/ml LPS (Fig. 3). Thus, autophagy appears to be suppressed below the basal level in LPS-stimulated cells. A concentration-dependent increase in HIF1α levels was observed 6 h after LPS stimulation, but the HIF1α levels were downregulated significantly 24 h after 10–100 ng/ml LPS stimulation (Fig. 3). This downregulation of HIF1α levels was not observed 12 h after LPS stimulation, suggesting that the downregulation takes place between 12 and 24 h (Supplementary Fig. 3). Corresponding to the downregulation of HIF1α, PKM2, a coactivator HIF1α^18^, was also downregulated in cells stimulated with 10–100 ng/ml LPS for 72 h. Taken together, autophagy is downregulated during LPS administration. During LPS treatment, HIF1α is upregulated at 6 h, but downregulated at 24 h of stimulation.

### Relative levels of intracellular metabolites in RAW264.7 cells stimulated with LPS

**Figure 4:**
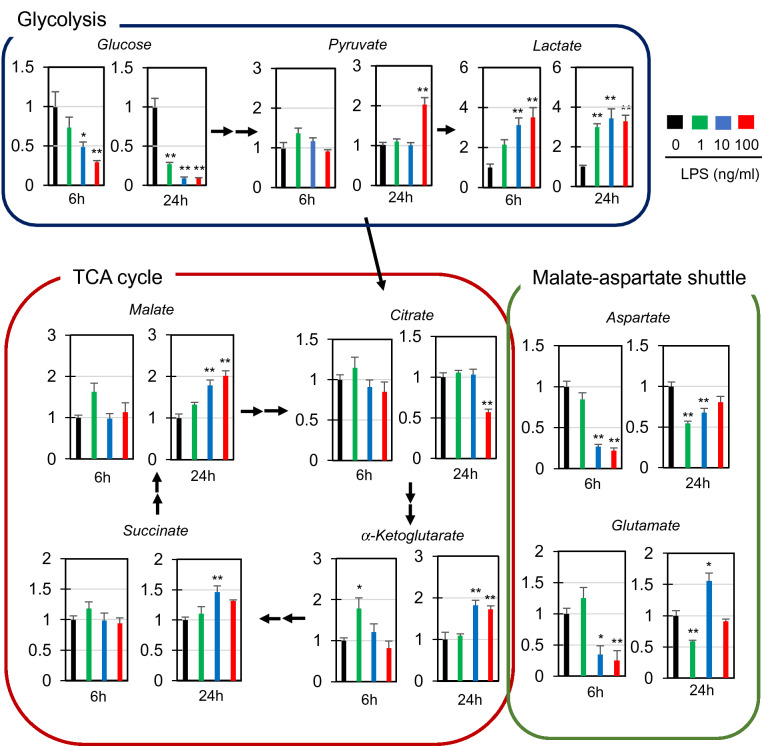
Comparative analysis of several metabolites involved in glycolysis, the TCA cycle, and the malate-aspartate shuttle in RAW264.7 cells after treatment with LPS. The cells were treated with the indicated concentrations of LPS for 6 or 24 h. Total cell lysates were extracted, and the levels of the indicated metabolites were examined by GC-MS. The data represent means and S.E., n = 3 or 4, *P < 0.05, **P < 0.01 (one way ANOVA followed by Dunnett’s post-hoc multiple comparison test).

To obtain further evidence of the metabolic shift toward glycolysis in LPS-stimulated cells, we examined the relative levels of several metabolic intermediates involved in glycolysis and the TCA cycle in LPS-stimulated RAW264.7 cells. Cells were stimulated with 1, 10, or 100 ng/ml LPS for 6 and 24 h, and cell extracts were analyzed by GC-MS. A greater than threefold increase in intracellular lactate levels was observed in LPS-stimulated cells as compared to non-stimulated cells (Fig. 4). Correspondingly, intracellular glucose levels dropped to 20–30% (1 ng/ml) and approx. 10% (10 and 100 ng/ml) in LPS (24 h)-stimulated cells as compared to those in non-stimulated cells (Fig. 4). Expression of a glucose transporter (GLUT1), which is involved in the metabolic shifting of macrophages^26^, was induced by LPS within 6 h and sustained for at least 24 h (Supplementary Fig. 4). Thus, the drop in intracellular glucose levels in response to LPS stimulation should result from an increased conversion of glucose to glucose-6-phosphate due to the increase in glycolysis. In cells stimulated with 10–100 ng/ml LPS, we observed significant decreases in aspartate and glutamate after 6 h of stimulation, as well as significant increases in α-ketoglutarate and malate after 24 h of stimulation (Fig. 4). Since these four metabolites are components of the malate-aspartate shuttle that transports glycolysis-derived NADH to the mitochondrial respiratory complex across the mitochondrial membrane, the balance between glycolysis and oxidative phosphorylation appears to be altered in LPS-stimulated cells as compared to non-stimulated cells. These results confirm that LPS stimulation leads to an M1 phenotype, which is associated with enhanced glycolysis and impaired oxidative phosphorylation. It should be noted that we also observed a slight but significant increase in succinate (Fig. 4), which has been shown to be upregulated in macrophages activated in the pro-inflammatory direction^27^.

### Methylglyoxal accumulates in LPS-stimulated RAW264.7 cells, and is involved in the downregulation of HIF1α

**Figure 5:**
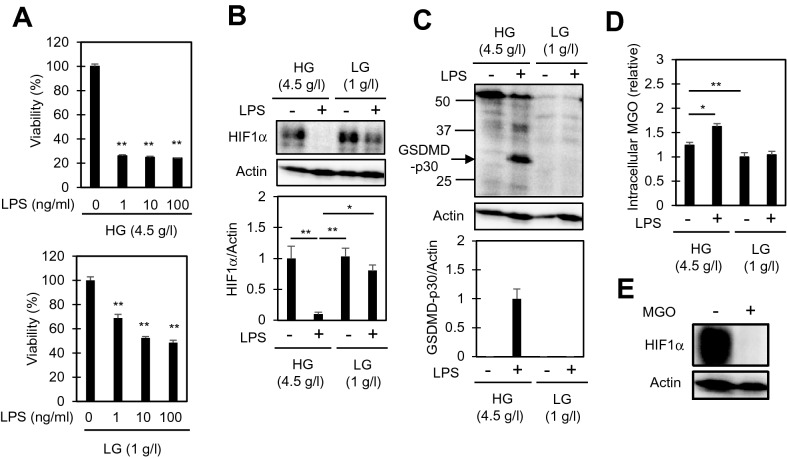
Pyroptosis in LPS-stimulated RAW264.7 cells is not observed under low glucose conditions. (**A**) Comparison of the rates of cell death between high and low glucose supplementation. The cells were treated with the indicated concentrations of LPS for 48 h in media containing high (4.5 g/l, HG) or low (1 g/l, LG) concentrations of glucose. Cell viabilities were determined by CCK8 assay. The mean viability of the control group (0 ng/ml LPS) was set as 100%. The data represent means and S.E., n = 4, **P < 0.01 (one-way ANOVA followed by Dunnett’s post-hoc multiple comparison test). (**B**, **C**) Immunoblot analysis of HIF1α and GSDMD in cells treated with the indicated concentrations of 10 ng/ml LPS for 24 (**B**) or 48 (**C**) hours. Actin was also examined as an internal standard. (**D**) Intracellular levels of methylglyoxal (MGO) in cells treated with 10 ng/ml LPS for 24 h. Total cellular lysates were extracted, and the MGO levels were examined by LC-MS. The mean level of the control group (0 ng/ml LPS, LG) was set as 100%. The data represent means and S.E., n = 4, *P < 0.05, **P < 0.01 (one-way ANOVA followed by Tukey–Kramer post-hoc multiple comparison test). (**E**) Extracellularly added MGO downregulates HIF1α. The cells were treated with 1 mM MGO for 1 h and the relative HIF1α levels were determined by immunoblotting.

Given the evidence that enhanced glycolysis leads to a drop in the intracellular glucose level within 24 h (Fig. 4), we examined the effect of altering the glucose concentration of the culture medium from 4.5 g/l (high glucose) to 1 g/l (low glucose). As shown in Fig. 5A, there was a smaller decrease in viability observed in LPS-stimulated cells cultured in low glucose medium as compared to high glucose medium. We next compared HIF1α as well as GSDMD between cells cultured in high and low glucose medium. As shown in Fig. 5B, C, no significant downregulation of HIF1α or GSDMD cleavage into p30 was observed in LPS-stimulated cells cultured in low glucose medium. These results suggest that the pyroptosis observed in LPS-stimulated cells cultured in high glucose medium did not result from a shortage of glucose. We also compared the mRNA and protein levels of LPS-induced IL-1β, and observed higher IL-1β expression in high glucose medium as compared to low glucose medium, confirming the pro-inflammatory properties of cells cultured in high glucose medium (Supplementary Fig. 5). Using LC-MS, we next examined the intracellular levels of methylglyoxal (MGO). MGO is a dicarbonyl compound generated as a by-product of glycolysis and the pentose phosphate pathway, and is highly reactive to proteins including HIF1^28^. MGO is also suggested as an exaggerating factor in hyperglycemia-aggravated illnesses^29^ including diabetes^28^, cardiac ischemia^30^, degenerative brain disorders^29^, and sepsis^31^. LPS significantly increased MGO levels only in cells cultured in high glucose medium (Fig. 5D). Immunoblot analysis using anti-MG-H1 antibody, which reacts with MGO-modified arginine residues in proteins, also showed increased MGO-modified protein levels in the cells cultured in high glucose medium (Supplementary Fig. 5). Furthermore, the addition of MGO to cells, which resulted in a significant increase in intracellular MGO levels (Supplementary Fig. 5), rapidly downregulated HIF1α (Fig. 5E), as reported previously^32^. The addition of iodoacetamide, a frequently-used inhibitor of glycolysis^33^, resulted in a significant increase in MGO, confirming that MGO is mainly produced from glycolytic intermediates (Supplementary Fig. 5). Collectively, the intracellular accumulation of MGO should be involved, at least in part, in the downregulation of HIF1α in LPS-stimulated cells cultured in high glucose medium.

### Suppression of pyroptosis by 2-deoxyglucose and succinate

**Figure 6:**
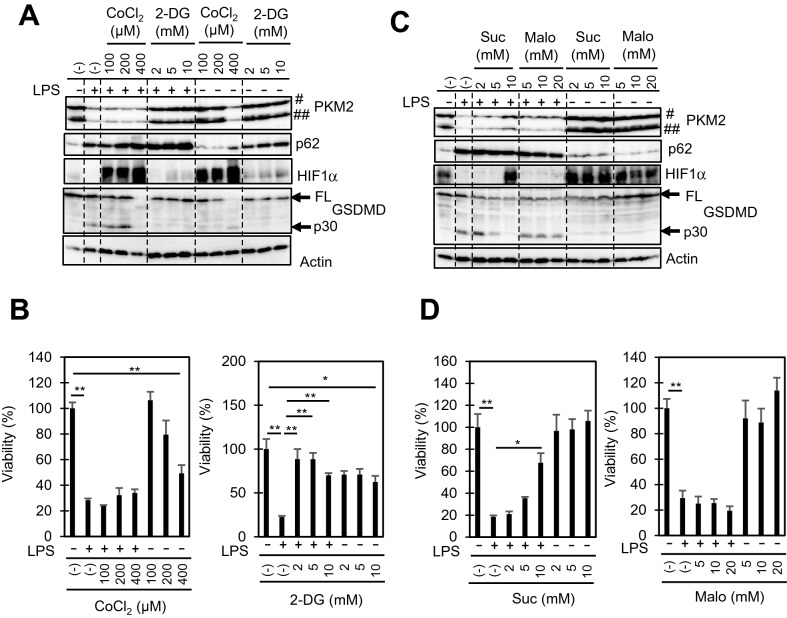
Effects of inhibitors and activators on LPS-stimulated RAW264.7 cells. The cells were pre-treated with the indicated concentrations of cobalt chloride (CoCl_2_), 2-deoxy-d-glucose (2-DG), diethyl succinate (suc), or dimethyl malonate (malo) for 30 min, and then treated with 10 ng/ml LPS for 48 h. (**A**, **C**) Total cellular lysates were extracted, and the levels of the indicated proteins were examined by immunoblotting. (**B**, **D**) Cell viabilities were also determined by CCK8 assay. Mean viability of the control group (0 ng/ml LPS) was set as 100%. The data represent means and S.E., n = 4, *P < 0.05, **P < 0.01 (one-way ANOVA followed by Tukey–Kramer post-hoc multiple comparison test).

We further examined the possible role of enhanced glycolysis as well as decreased oxidative phosphorylation in the downregulation of HIF1α and pyroptosis in RAW264.7 cells. CoCl_2_, an HIF1α activator, could not suppress pyroptosis in LPS-stimulated cells (Fig. 6A), and 400 µM CoCl_2_ showed significant cytotoxicity (Fig. 6B) that involved GSDMD cleavage into p30 (Fig. 6A). In contrast, the glycolysis inhibitor 2-deoxy-d-glucose (2-DG) suppressed all of the PKM2 and HIF1α downregulation, decrease in viability, and GSDMD cleavage (Fig. 6A, B). In addition, we found that succinate, which is involved in the activation of PKM2^34^ and HIF1α^35^, suppressed the downregulation of PKM2 and HIF1α as well as the decrease in viability and cleavage of GSDMD when applied at 10 mM to cells as a cell permeable analogue, diethyl succinate (Fig. 6C, D). In contrast, the complex II inhibitor diethyl malonate had no effect on LPS-stimulated cells (Fig. 6C, D). Taken together, the re-activation of HIF1α is not sufficient for the suppression of pyroptosis. In contrast, the inhibition of glycolysis and the addition of succinate are sufficient to prevent HIF1α downregulation as well as pyroptosis.

### Rapamycin suppresses pyroptosis

**Figure 7:**
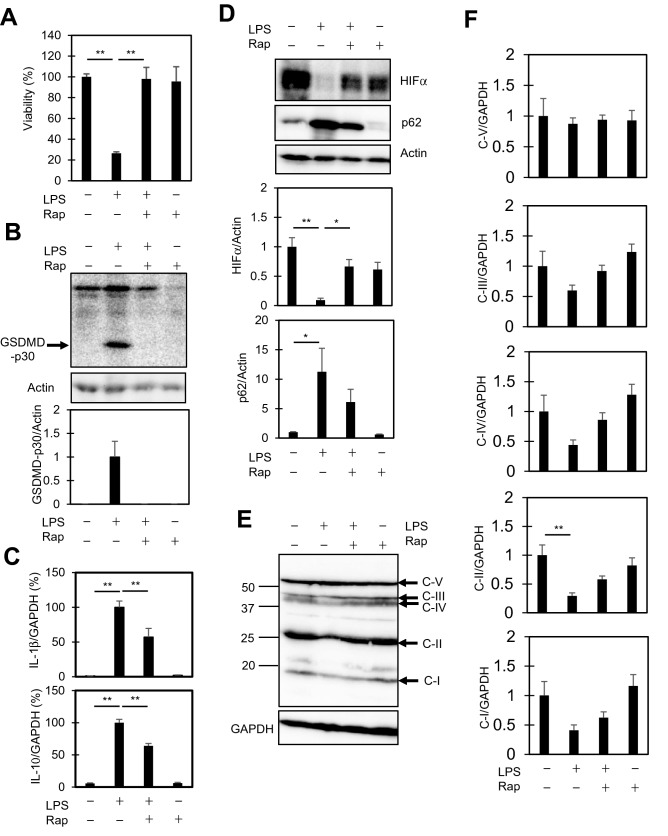
Effects of rapamycin on LPS-stimulated RAW264.7 cells. (**A**, **B**) Rapamycin inhibits pyroptosis. The cells were pre-treated with 10 µM rapamycin (rap) for 30 min, and then treated with 10 ng/ml LPS for 48 h. Cell viabilities were determined by CCK8 assay (**A**). Mean viability of the control group (0 ng/ml LPS) was set as 100%. Total cellular lysates were also extracted, and the levels of GSDMD-p30 were examined by immunoblotting. (**B**). (**C**) Rapamycin suppresses the induction of cytokine expression by LPS. qPCR analysis was performed to evaluate the levels of IL-1β and IL-10 relative to GAPDH. (**D**) Rapamycin suppresses downregulation of HIF1α. Immunoblot analysis of HIF1α and p62 is shown. (**E**) Effect of rapamycin on OXPHOS proteins. Immunoblot analysis of OXPHOS (**E**, **F**). The data represent means and S.E., n = 3 or 4, *P < 0.05, **P < 0.01 (one-way ANOVA followed by Tukey–Kramer post-hoc multiple comparison test).

Given the indication of decreased autophagy (Fig. 3) as well as increased glycolysis (Figs. 2, 4), we finally examined the effect of rapamycin on LPS-stimulated RAW264.7 cells. Rapamycin is an antibiotic that acts in various ways including as an immunosuppressant, autophagy activator, and glycolysis inhibitor^36^. Thus, rapamycin negatively regulates macrophage activation by inhibiting glycolytic metabolism and stimulating autophagy. The addition of rapamycin restored the viability that was lost in LPS-stimulated cells (Fig. 7A) and suppressed GSDMD-p30 formation (Fig. 7B). Moreover, rapamycin suppressed the inductions of both IL-1β and IL-10 (Fig. 7C), as well as the downregulation of HIF1α (Fig. 7D). Rapamycin also tended to prevent the downregulation of OXPHOS proteins in LPS-stimulated cells (Fig. 7E, F). The effectiveness of rapamycin on the suppression of IL induction, recovery from the downregulation of HIF1α, and suppression of pyroptosis further confirms that glycolysis is a key event not only in macrophage activation, but also in pyroptosis.

## Discussion

At first, we suspected metabolic acidosis as the primary cause of pyroptosis during LPS stimulation under high glucose supplementation. Indeed, LPS stimulation of RAW264.7 cells cultured in high glucose medium resulted in a significant increase in intracellular lactate within 24 h (Fig. 4). Further stimulation for 72 h led to a severe decline in the extracellular pH (~ 5.7). Since lactate is excluded from cells through monocarboxylate transporters (MCTs)^37,38^, the extracellular acidosis should be caused mainly by the excretion of intracellular lactate. A previous report demonstrated that acidosis itself could activate inflammasomes^39^. However, we observed neither a significant decrease in viability nor an increase in LDH release even in cells treated with up to 30 mM lactate (Supplementary Fig. 6). Thus, lactic acidosis seems not be the main cause of pyroptosis in LPS-stimulated RAW264.7 cells.

HIF1α activation is required for cells to maintain homeostasis under low-oxygen conditions. Indeed, in a low oxygen environment, HIF1α deficiency results in cell death^40^. The accumulation of MGO under hyperglycemia and the subsequent degradation of HIF1α are proposed as mechanisms underlying the detrimental effects of high glucose on various injuries,^41^ such as hypoxic cardiomyocyte injuries^42^. MGO modifies HIF1α at its proline residues, leading to ubiquitination by the C-terminus of the HSC70-interacting protein (CHIP) and subsequent proteasome-dependent degradation^32^. Therefore, our current findings that intracellular MGO levels not only correlate with the induction of pyroptosis, but also inversely correlate with the levels of HIF1α (Fig. 5B, C) imply that the detrimental effect of high glucose on LPS-induced RAW264.7 cells might be derived, at least in part, from the MGO-dependent degradation of HIF1α. HIF1α downregulation has also been reported in cells during prolonged hypoxia^43^. Interestingly, the downregulation of HIF1α by prolonged hypoxia is also mediated by CHIP^44^. Thus, the downregulation of HIF1α below the basal level that is observed in LPS-stimulated cells (Fig. 3) might result from an enhanced downregulation by CHIP.

Although CoCl_2_ caused the upregulation of HIF1α, it did not suppress pyroptosis in LPS-stimulated cells (Fig. 6A, B). Rather, CoCl_2_ alone seemed to induce GSDMD cleavage into p30 when applied at a concentration of 400 µM (Fig. 6A). It should be noted that CoCl_2_ did not upregulate PKM2 levels even though it upregulated HIF1α (Fig. 6A). In contrast to CoCl_2_, 2-DG not only suppressed the downregulations of HIF1α and PKM2, but also ameliorated GSDMD cleavage and the loss of viability in LPS-stimulated cells (Fig. 6A, B). Thus, the re-activation of HIF1α is not sufficient to inhibit pyroptosis. Other molecules such as PKM2 might be required for the transcriptional activation of HIF1, as well as survival during prolonged LPS stimulation in macrophages.

The mitochondrial complex II inhibitor diethyl malonate had no effect on any of the LPS-induced changes tested, while dimethyl succinate suppressed all of the downregulations of HIF1α as well as PKM2, GSDMD cleavage, and loss of viability, when applied at a concentration of 10 mM (Fig. 6C, D). Succinate has been shown to accumulate during the proinflammatory activation of macrophages, and is believed to act as a mediator of HIF1α activation^27,45^. In contrast, malonate has been shown to suppress LPS-induced HIF1α activation^46^. Therefore, our finding that succinate reactivates HIF1α while malonate does not is in agreement with the previous reports. However, our results also indicate that succinate acts to suppress pyroptosis (Fig. 6C, D). The role of succinate in macrophage activation might differ between the early and late stages of pyroptosis.

In conclusion, we show that LPS stimulation of RAW264.7 macrophages in high glucose medium leads to pyroptosis, during which HIF1α is downregulated to below the basal level. This downregulation of HIF1α is caused, at least in part, by the cellular accumulation of MGO in LPS-stimulated cells cultured in high glucose medium. These results reveal that pyroptosis, like the inflammasome, is crucially regulated by glycolysis.

## Experimental procedures

### Materials

Lipopolysaccharides (LPS, *E. coli* O111:B4, L2630), methylglyoxal (MGO, M0252), cobalt chloride (CoCl_2_, C8661), 2-deoxy-D-glucose (2-DG, D6134), diethyl succinate (suc, W237701), dimethyl malonate (malo, 136441), and rapamycin (rap, R0395) were purchased from Sigma-Aldrich (St. Louis, MO).

### Cell culture

The RAW264.7 murine macrophage/monocyte cell line was obtained from RIKEN (RCB0535, Tsukuba, Japan), and maintained in DMEM (high glucose, 4.5 g/l) supplemented with 10% heat-inactivated FBS and antibiotics (streptomycin sulfate and penicillin at final concentrations of 100 U/ml and 100 µg/ml, respectively) at 37 °C under a 5% CO_2_ atmosphere. During stimulation with LPS, cells were cultured in 1 ml of high glucose DMEM containing FBS on 3.5 diameter dishes. In some experiments, low glucose (1 g/l) DMEM was used instead of high glucose DMEM. The pH of the medium was measured by a portable pH meter (LAQUAtwin, Horiba, Tokyo, Japan).

### Determination of cell death and cell viability

The percentages of LDH released into the medium were measured by the LDH-Cytotoxic Test (299-50601, Wako, Osaka, Japan). Cell viability was evaluated using a Cell Counting Kit-8 (CCK-8, CK04, Dojindo, Kumamoto, Japan).

### Quantitative reverse transcriptase-mediated real-time PCR (qPCR)

Total RNA was extracted from the cells using Trizol reagent (Thermo Fisher Scientific, Waltham, MA). After extraction of total RNA, complementary DNA (cDNA) was synthesized using oligo (dT)_15_ as a primer and SuperScript II as a reverse transcriptase (RT, Thermo Fisher Scientific). RT-mediated real-time PCR (RT-PCR) was performed using the StepOnePlus system (Thermo Fisher Scientific) that uses SYBR Green as a dye (GoTaq qPCR master mixture, Promega, Madison, MI). The relative abundance of mRNAs was calculated by the comparative Ct method. Primers used are listed in Supplementary Table 1.

### Immunoblotting

Total cellular lysates were extracted from the cells, separated by SDS-PAGE, and transferred to a PVDF membrane. After blocking with 3% milk, blots were probed with primary antibodies (Supplementary Table 2) and HRP-conjugated secondary antibodies (Promega), developed with an ECL system, and images were captured by image capture (LuminoGraph III, ATTO, Tokyo, Japan). ImageJ (1.47v) was used to quantify band intensities.

### GC-MS analysis of metabolites

Cells grown on 3.5 cm diameter dishes were collected in PBS, and centrifuged at 1,000 rpm for 1 min at 4 °C. The cell pellets were disrupted by ultrasonication in 500 µl methanol, followed by further centrifugation at 15,000 rpm for 15 min at 4 °C. After removing the supernatants, water (100 µl) was added to the pellets to extract the highly hydrophilic metabolites and added to the supernatants (methanol solution). 2-Isopropylmalic acid was added as an internal standard. Metabolites were dried by vacuum evaporation, dissolved in 100 µl pyridine containing 20 mg/ml methoxyamine, and incubated at 37 °C for 90 min. After trimethylsilylation with MSTFA (*N*-methyl-*N*-trimethylsilyltrifluoroacetamide), the metabolites were subjected to GC-MS analysis (GCMS-TQ8030, Shimadzu, Kyoto, Japan).

### LC-MS analysis of methylglyoxal

The measurement of methyglyoxal was performed based on the published method^47,48^. In brief, cell pellets (from 3.5 cm diameter dishes) were dissolved in trichloroacetic acid (TCA)-saline solution and further incubated with *O*-phenylenediamine to convert methylglyoxal into 2-methylquinoxaline (2-MQ). Detection of derivatized methylglyoxal, 2MQ, was performed with multiple reaction monitoring (MRM) analysis using LC-MS (LCMS-8040, Shimadzu). MRM mass transition and collision energy (eV) were 145.1 > 77.1, 24 and 145.1 > 92.1, 20. The intracellular level of methylglyoxal in the cells was estimated by the standard addition method.

### Statistical analysis

For statistical analysis, GraphPad Instat (Version 3.1a, GraphPad Software, Inc., La Jolla, CA) was used. *P* < 0.05 was considered statistically significant.

## Supplementary information

Supplementary file1 (DOCX 23551 kb)
