# Supplementary material for: Extracellular glucose is crucially involved in the fate decision of LPS-stimulated RAW264.7 murine macrophage cells
Source: Sci Rep. 2020 Jun 29;10:10581. doi: 10.1038/s41598-020-67396-6 (PMC7324593; doi:10.1038/s41598-020-67396-6)

**Supplementary Information**

**Extracellular glucose is crucially involved in the fate decision of LPS-stimulated RAW264.7 murine macrophage cells**

Toshihiko Aki^1^, Takeshi Funakoshi^1^, Kanako Noritake^1^, Kana Unuma^1^, and Koichi Uemura^1^

^1^ Department of Forensic Medicine, Graduate School of Medical and Dental Sciences, Tokyo Medical and Dental University, Tokyo Japan

**Supplementary Table1. Primers used in this study**

| Gene | Forward | Reverse |
| --- | --- | --- |
| IL-1β | TGCCACCTTTTGACAGTGATG | TGATGTGCTGCTGCGAGATT |
| IL18 | ACTTTGGCCGACTTCACTGT | GTCTGGTCTGGGGTTCACTG |
| IL10 | AGGCGCTGTCATCGATTTCT | ATGGCCTTGTAGACACCTTGG |
| iNOS | GGTGAAGGGACTGAGCTGTT | TGCACTTCTGCTCCAAATCCA |
| Arg1 | GGGACCAGGTGCTAATCCCT | CTGCAGTTTCCAAACAGGGC |
| GLUT1 | TGGCGGGAGACGCATAGTTA | CTCCCACAGCCAACATGAGG |
| GAPDH | GTGCAGTGCCAGCCTCGTCC | AACGCAGCTCAGTAACAGTCC |

**Supplementary Table2. Antibodies used in this study**

| Antibody | Source | Identifier |
| --- | --- | --- |
| Anti-IL-1β | R&D Systems | AF-401-NA |
| Anti-IL-18 | MBL | DO46-3 |
| Anti-LDH | abcam | ab52488 |
| Anti-cleaved caspase3 | Cell Signaling | #9661 |
| Anti-GSDMD | Santa Cruz | sc-393656 |
| Anti-DFNA5 | abcam | ab215191 |
| Anti-OXPHOS | abcam | Ab110413 |
| Anti-GAPDH | Millipore | MAB374 |
| Anti-LC3 | Cell Signaling | #2775 |
| Anti-p62 | MBL | PM045 |
| Anti-HIF1α | Cell Signaling | #36169 |
| Anti-PKM2 | Cell Signaling | #3198 |
| Anti-actin | Sigma-Aldrich | A2066 |
| Anti-MG-H1 | Novus Biologicals | NBP2-62810 |

**Supplementary Fig. 1**


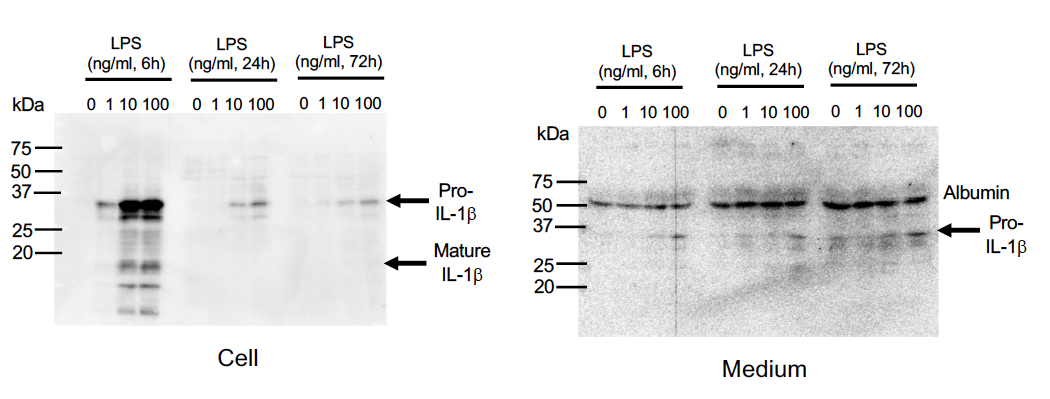


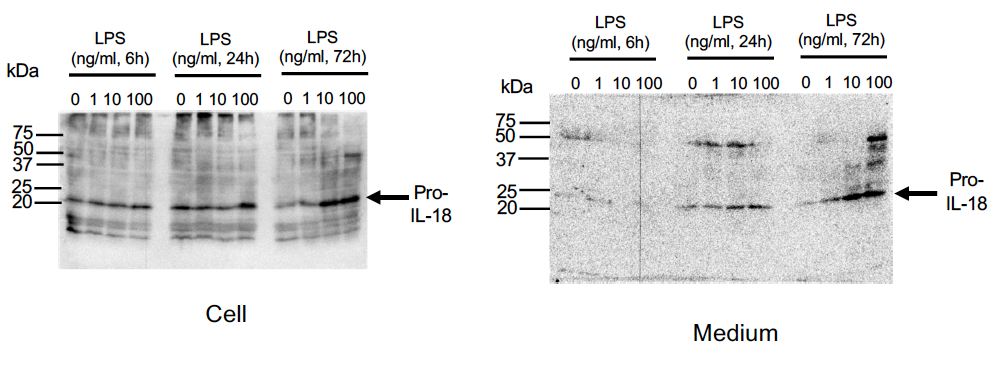


Protein levels of IL-1β and IL-18 in RAW264.7 cells after treatment with LPS. The cells were treated with the indicated concentrations of LPS for the indicated time periods. Total cell lysates (cell) and conditioned medium (medium) were used for immunoblot analysis.

**Supplementary Fig. 2**


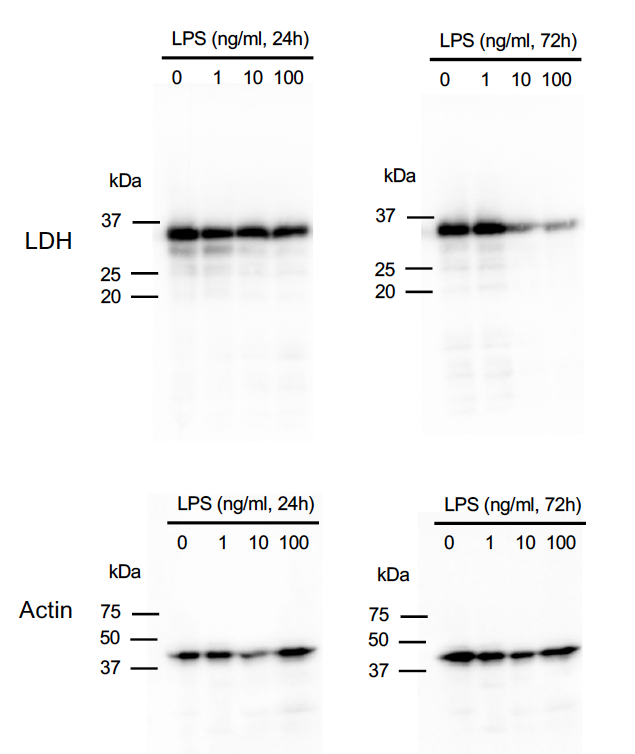


Levels of LDH in RAW264.7 cells after treatment with LPS. The cells were treated with the indicated concentrations of LPS for the indicated time periods. Total cell lysates were used for immunoblot analysis.

**Supplementary Fig. 3**


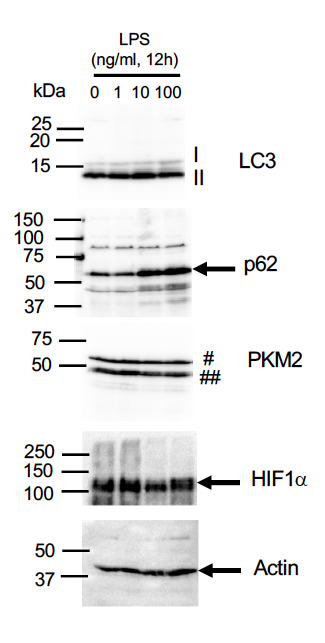


Levels of autophagy markers, PKM2, and HIF1α in RAW264.7 cells after treatment with LPS for 12 hours. Total cell lysates were extracted, and the levels of the indicated proteins (LC3, p62, HIF1α, PKM2, and actin) were determined by immunoblotting. #, a band corresponding to the molecular weight of PKM2 (~60 kDa).

**Supplementary Fig. 4**


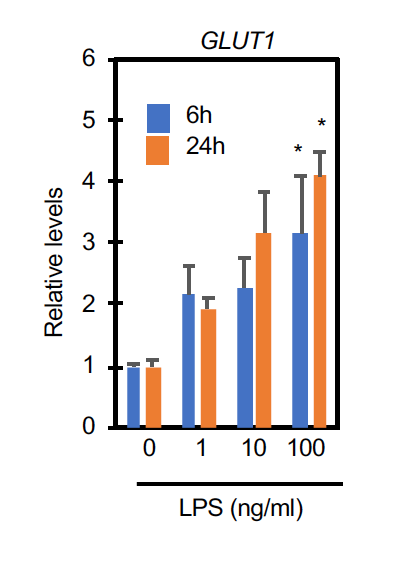


Expression of GLUT1 in RAW264.7 cells stimulated by LPS. The cells were treated with the indicated concentrations of LPS for 6 and 24 hours, and qPCR analysis was performed to evaluate the levels of GLUT1 relative to GAPDH. The data represent mean and S.E., n=4, *p < 0.05 versus 0 ng/ml (one-way ANOVA followed by Dunnett’s post-hoc multiple comparison test).

**Supplementary Fig. 5**


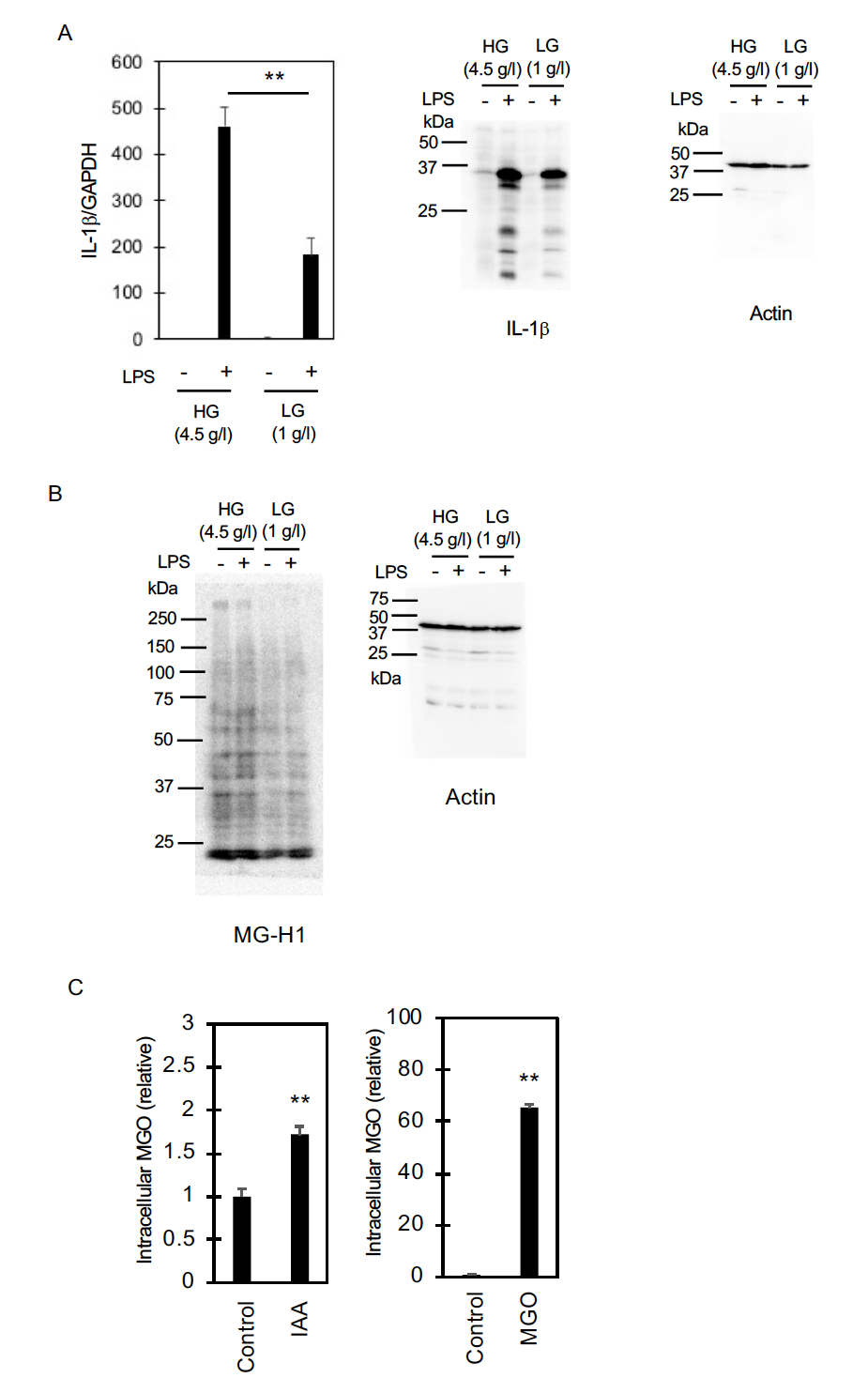


(A) Expression of IL-1β in RAW264.7 cells stimulated by LPS. The cells were treated with 10 ng/ml LPS for 6 hours in media containing high (4.5 g/l, HG) or low (1 g/l, LG) concentrations of glucose. qPCR analysis was performed to evaluate the levels of IL-1β, relative to GAPDH (left panel). The data represent mean and S.E., n=4, **p < 0.01 (one-way ANOVA followed by Tukey-Kramer post-hoc multiple comparison test). Immunoblot analyses were also performed to evaluate the levels of IL-1β and actin (middle and right panels).

(B) Levels of MGO-modified proteins in RAW264.7 cells after treatment with LPS (10 ng/ml) for 24 hours. Total cell lysates were extracted, and the levels of the MGO-modified proteins were determined by immunoblotting using MG-H1 antibody.

(B and C) Intracellular levels of methylglyoxal (MGO) in cells treated with 1 mM iodoacetamide (IAA) for 30 min (B) or cells treated with 1 mM MGO for 1 hour (C). Total cellular lysates were extracted and the levels of MGO were examined by LC-MS. The mean level of the control group was set as 1. The data represent mean and S.E., n=4, **p < 0.01 (Student’s *t-*test).

**Supplementary Fig. 6**


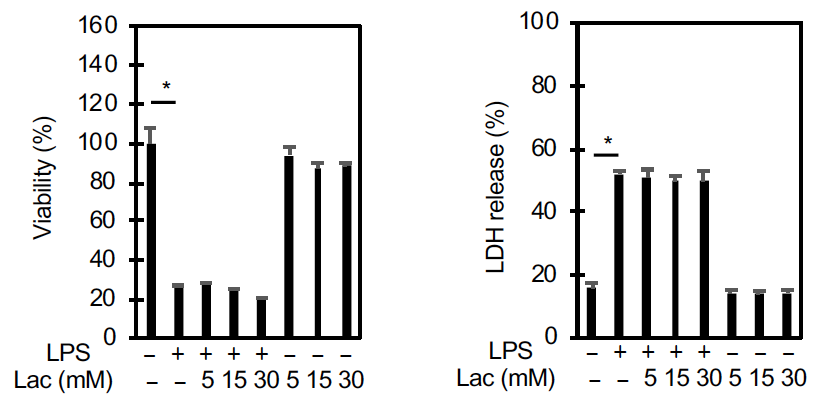


Effects of lactate on LPS-stimulated RAW264.7 cells. The cells were pre-treated with the indicated concentrations of lactate (Lac) for 30 min, and then treated with 10 ng/ml LPS for 48 hours. LDH release assay and CCK8 assay were performed. Mean cellular LDH activity and viability of control group (0 ng/ml LPS) were set as 100%. The data represent mean and S.E., n=4, *p < 0.05, **p < 0.01 (one-way ANOVA followed by Tukey-Kramer post-hoc multiple comparison test).

**Supplementary Fig. 7**

The full length blots of Fig. 2


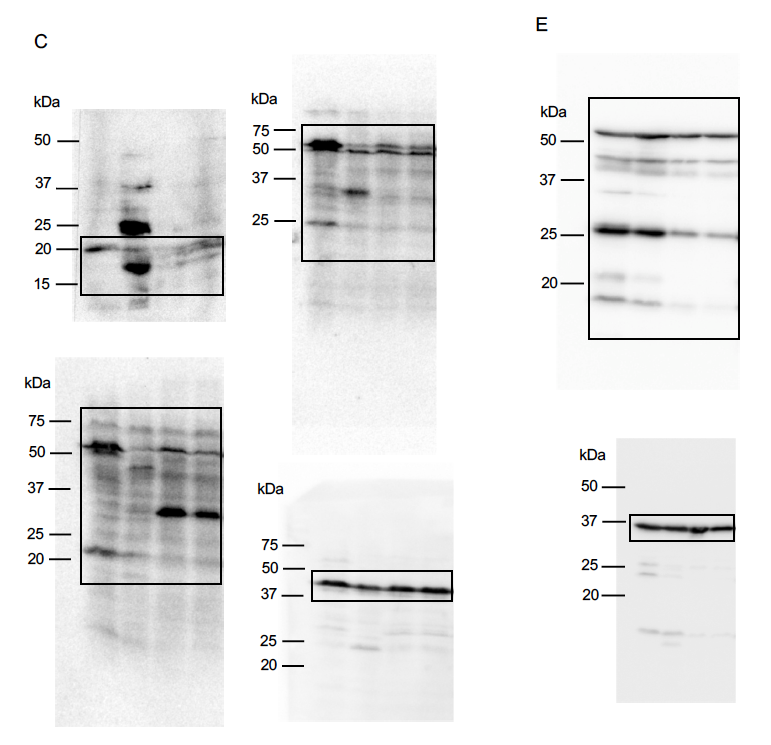


The full length blots of Fig. 3

**
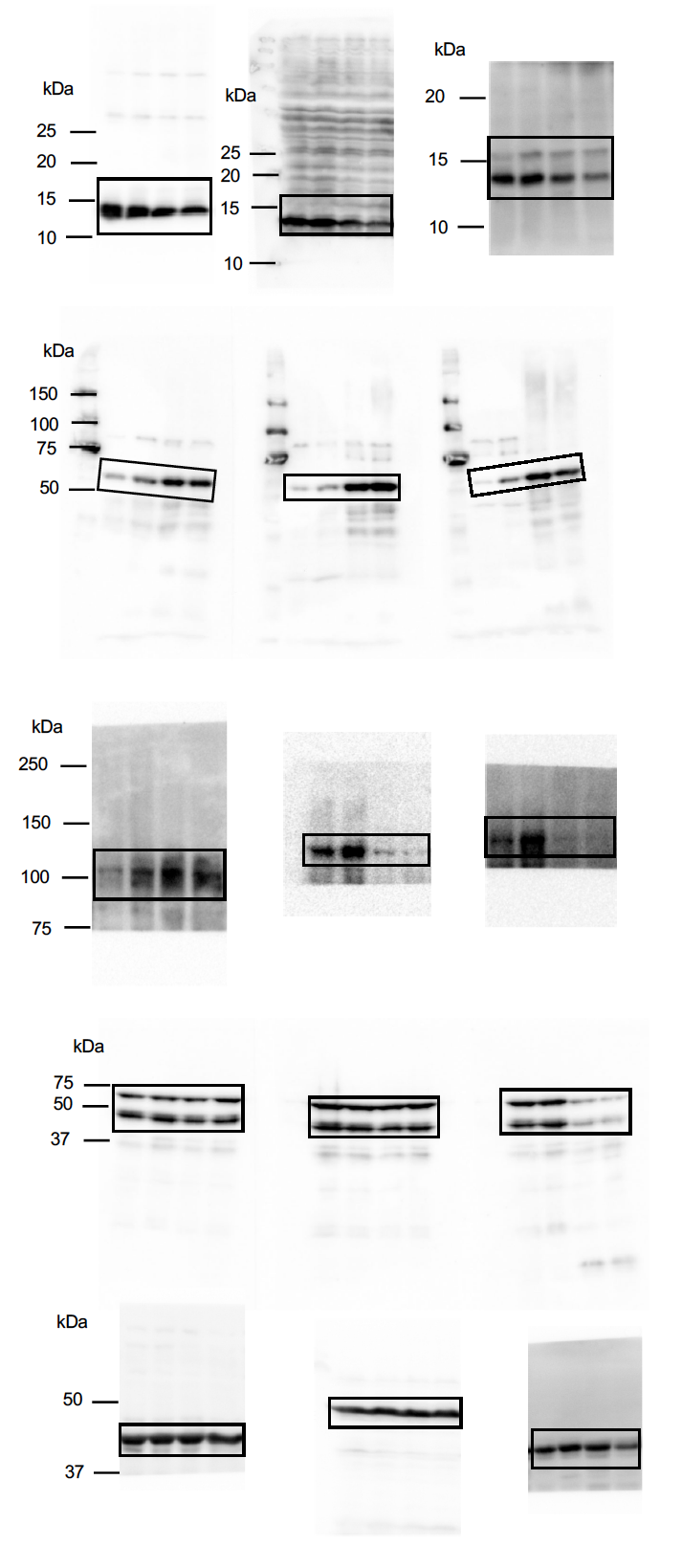
**

The full length blots of Fig. 5

**
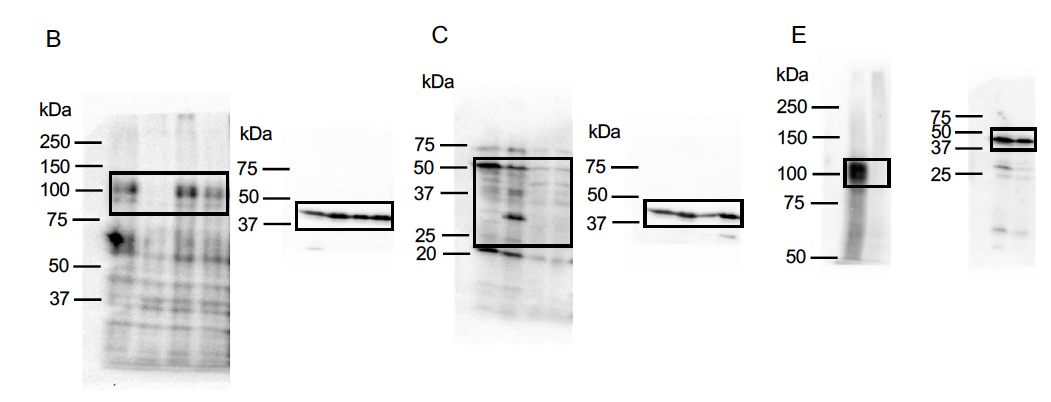
**

The full length blots of Fig. 6


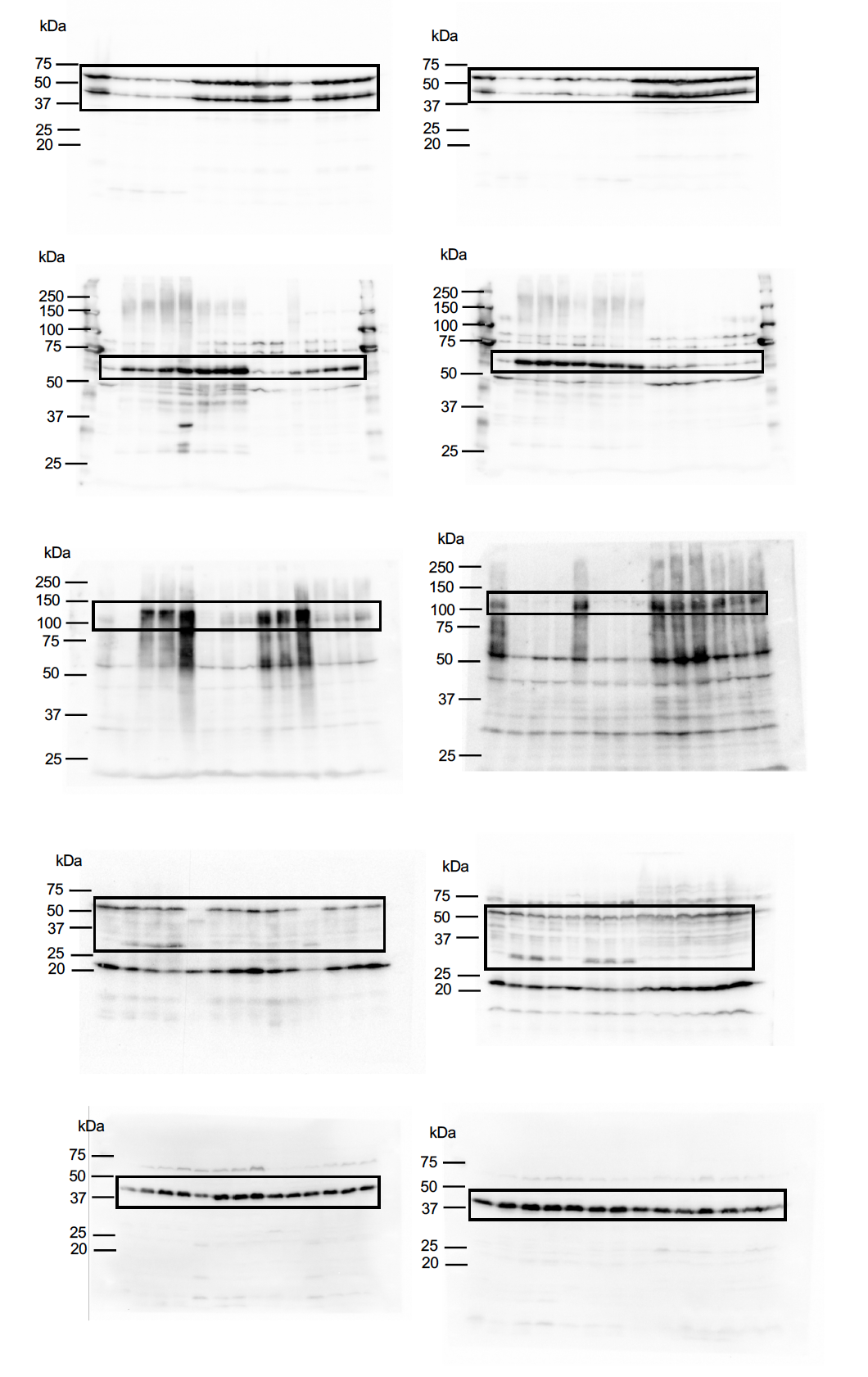


The full length blots of Fig. 7


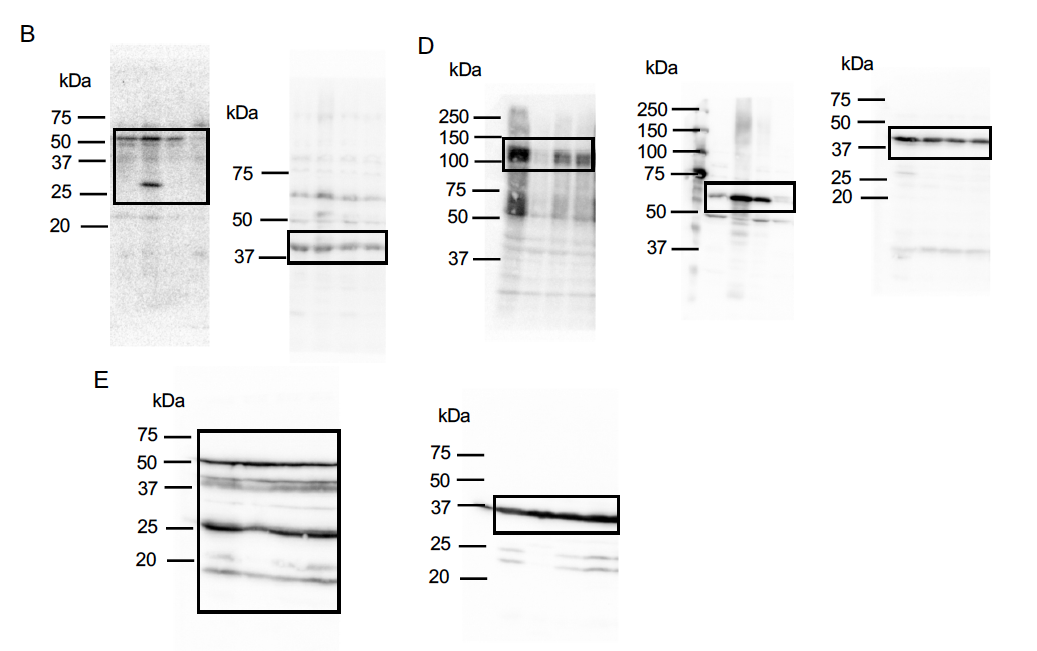

Supplement: Supplementary file 1 — Supplementary file1 (DOCX 23551 kb) [file 41598_2020_67396_MOESM1_ESM.docx]
